# *Sheathia shaoguanensis* sp. nov. (Batrachospermales, Rhodophyta), a new freshwater red algal species from South China

**DOI:** 10.3897/phytokeys.277.198746

**Published:** 2026-07-06

**Authors:** Jin-fen Han, Xin Wang, Bin Yin, Shu-lian Xie

**Affiliations:** 1 College of Architecture and Design, Shanxi Vocational University of Engineering Science and Technology, Jinzhong 030619, China School of Life Science, Shanxi Key Laboratory for Research and Development of Regional Plants, Shanxi University Taiyuan China; 2 School of Engineering Management, Shanxi Vocational University of Engineering Science and Technology, Jinzhong 030619, China College of Architecture and Design, Shanxi Vocational University of Engineering Science and Technology Jinzhong China; 3 School of Life Science, Shanxi Key Laboratory for Research and Development of Regional Plants, Shanxi University, Taiyuan 030006, China School of Engineering Management, Shanxi Vocational University of Engineering Science and Technology Jinzhong China

**Keywords:** New species, phylogeny, *Sheathia* sp., unique habitat

## Abstract

The freshwater red algal order Batrachospermales has a triphasic life history, of which the morphological characteristics of “Chantransia” stages are very similar to those of the genus *Audouinella*. In this study, two gametophyte stage specimens (CQM21 and CQW23) and four “Chantransia” stage specimens (CQ003, CQM22, CQW24, and GD247) were collected from Chongqing Municipality and Guangdong Province in China. Based on morphological data, all four “Chantransia” isolates were similar to *A. pygmaea*, whereas the morphological characteristics of CQM21 and CQW23 were in good agreement with the circumscription description of *Sheathia dispersa*. The *rbc*L and COI-5P analyses suggested that all six specimens belong to the genus *Sheathia* and supported the proposal of a new species, *S. shaoguanensis***sp. nov**. Nevertheless, the five specimens from Chongqing (including the two gametophyte stage and three “Chantransia” stage specimens) were all assigned to *S. dispersa*. This represents the first report of the genus *Sheathia* in the Yangtze River.

## Introduction

The freshwater red algal genus *Sheathia* Salomaki & M.L.Vis (Batrachospermales, Rhodophyta) belonging to the order Batrachospermales (Florideophyceae, Rhodophyta) was elevated from section *Helminthoidea* based on morphological and molecular phylogenetic studies by Salomaki et al. (2014). To date, species of the genus *Sheathia* are widely reported in temperate, sub-Arctic, and subtropical freshwater systems (Salomaki et al. 2014; Necchi et al. 2019; Kitayama and Suzuki 2024). It is one of the most abundant genera in the order Batrachospermales, with a total of 24 taxa reported (Guiry and Guiry 2022). Many morphological characteristics of this genus overlap significantly with the Sect. *Batrachospermum* of the genus *Batrachospermum* Roth. According to Salomaki et al. (2014), some species within the genus *Sheathia* exhibit heterocortication, a distinctive anatomical feature in which the cortex contains two morphologically distinct cell types: ‌spherical‌ and ‌cylindrical‌ cortical cells. This unique morphological structure serves as a key diagnostic character distinguishing *Sheathia* from other genera in the Batrachospermales. However, more than half of the species that have been reported so far lack the heterocortication feature and can only be distinguished based on molecular phylogenetic data, which brings difficulties to the classification and identification of this genus. For example, based on molecular sequences, *S. arcuata* (Kylin) Salomaki & M.L.Vis, *S. longipedicellata* (D.Hua & Z.X.Shi) J.F.Han & al., *S. dispersa* Necchi, J.A.West, E.K.Ganesan & S.K.Rai, *S. abscondita* Stancheva, Sheath & M.L.Vis, *S. assamica* Necchi, J.A.West, E.K.Ganesan & Yasmin, *S. californica* Stancheva, Sheath & M.L.Vis, *S. indonepalensis* Necchi, J.A.West, E.K.Ganesan, S.K.Rai & F.Yasmin, *S. murpheyi* A.L.Szinte, J.C.Taylor & M.L.Vis, *S. plantuloides* M.L.Vis, and *S. transpacific* M.L.Vis all belong to the genus *Sheathia*, but no obvious heterocortication were observed in their cortical cells (Hua and Shi 1996; Vis et al. 2020; Salomaki et al. 2014; Necchi et al. 2019).

Species within the order Batrachospermales generally exhibit a complex heteromorphic alternation of generations (Sheath 1984). The genus *‌Sheathia‌* follows this evolutionary pattern, with its complete life cycle encompassing three developmentally distinct stages characterized by marked morphological and functional differentiation: the gametophyte, carposporophyte, and a “Chantransia” asexual reproduction stage. The orderly alternation of these three stages is not only an important evolutionary strategy for *Sheathia* spp. to adapt to freshwater habitats, but also reflects the high degree of evolutionary conservation in the life cycle among Batrachospermales species, which provides an important research model for further understanding the phylogeny and ecological adaptability of Rhodophyta. The haploid gametophyte is the dominant life stage of the genus *Sheathia*, and its morphology is relatively complex. Most species within this genus are described and identified based on samples from this stage. In contrast, ‌the diploid “Chantransia” stage is microscopic and structurally simple‌, exhibiting indistinct species characteristics and easily confused with species from the genus *Audouinella* (order Acrochaetiales), thus requiring molecular data for accurate taxonomic identification (Han et al. 2020; Kitayama and Suzuki 2024). Two gametophyte stage specimens and four “Chantransia” stage specimens were collected in this study. Based on morphological observations and molecular phylogenetic analyses, we identified and proposed a new species (*S. shaoguanensis* sp. nov.) and a newly recorded species (*S. dispersa*) from China.

## Materials and methods

The algal materials were collected from Chongqing and Guangdong Province in China (Table 1). Samples were immediately fixed in 4% formalin solution for morphological observations. For these two life history stages, we measured all previously reported morphometric features (Tables 2, 3). Voucher specimens were deposited in the herbarium of Shanxi Vocational University of Engineering Science and Technology (GKD). Voucher information is detailed in Table 1. For observations and measurements, we used a BX-51 Olympus microscope equipped with a charge-coupled device (DP72; Olympus, Tokyo, Japan).

**Table 1.** Sample information and GenBank accession numbers of samples used in this study.

**Isolate**	**Locality with GPS coordinates**	**Voucher number**	***rbc*L**	**COI-5P**	**Species**
CQ003	Fengjie, Chongqing, China (31°01'N, 109°28'E)	CQ21053	PZ319263	PZ321415	*S. dispersa*
CQM21	Fengjie, Chongqing, China (31°01'N, 109°28'E)	CQ21521	PZ319264	PZ321416	*S. dispersa*
CQM22	Fengjie, Chongqing, China (31°01'N, 109°28'E)	CQ21522	PZ319265	PZ321417	*S. dispersa*
CQW23	Fengjie, Chongqing, China (31°01'N, 109°28'E)	CQ21523	PZ319266	PZ321418	*S. dispersa*
CQW24	Fengjie, Chongqing, China (31°01'N, 109°28'E)	CQ21524	PZ319267	PZ321419	*S. dispersa*
GD247	Guangdong Grand Canyon, Shaoguan, Guangdong Province, China (24°31'N, 113°07'E)	GD20247	PZ319268	PZ321420	*S. shaoguanensis* sp. nov.

**Table 2.** Comparison of the morphological characteristics among “Chantransia” stage specimens in this study, *S. yedoensis* and *A. pygmaea*.

**Thalli characteristics**	**CQ003**	**CQM22**	**CQW24**	**GD247**	***S. yedoensis* ^1)^**	***A. pygmaea* ^2)^**
Color	Brownish	Brownish	Brownish	Brownish	Reddish to dark brown	Reddish
Height (mm)	3.6–11.0	2.9–7.9	3.2–5.0	3.6–9.8	≤4	3.0–5.0
Branch angle^2)^	<25°	≤ 25°	<25°	≤ 25°	2°–21°	≥25°
Vegetative cells Length (μm)	33.3–77.8	24.4–68.9	28.9–57.8	22.1–33.7	14–57	26.4–50.7
Vegetative cells Diameter (μm)	6.7–15.6	8.9–17.8	8.9–15.6	5.3–7.4	8–10	12.2–15.2
Monosporangia Shape	Ovoid	Ovoid	Ovoid	Ovoid	Obovoidal to ellipsoidal	–
Monosporangia Length (μm)	11.1–20.0	15.6–20.0	15.6–21.1	8.4–13.7	10–14	8.9–13.0
Monosporangia Diameter (μm)	8.9–13.3	8.9–13.3	11.1–13.3	6.3–11.6	7–11	7.1–10.0

^1)^ Definition follows Kitayama and Suzuki (2024). ^2)^ Definition follows Necchi and Zucchi (1995).

**Table 3.** Comparison of the morphological characteristics among the gametophyte specimens examined in this study and those previously reported for *S. dispersa*.

**Taxon**	**CQM21 (male plant)**	**CQW23 (female plant)**	*Sheathia dispersa* ^*^
Whorl shape	Spherical- to barrel-shaped	Spherical	Barrel-shaped or spherical
Whorl diameter (μm)	409-545	455–709	483–1316
Carpogonial branch (cell number)	–	3–5	3–8
Carpogonium diameter (μm)	–	5.6–6.7	7.8–12.2
Carpogonium length (μm)	–	8.9–13.3	13.0–20.0
Trichogyne shape	–	Clavate	Clavate
Spermatangia diameter (μm)	5.5–7.3	–	5.8–8.1
Carposporophyte shape	–	Spherical	Spherical or subspherical
Carposporophyte diameter (μm)	–	103–353	97–235
Carposporophytes number per whorl	–	1–3	1–5
Carposporophytes position	–	Outer whorls	Within the whorls or exerted

^*^Definition follows Necchi et al. (2019).

For DNA analysis, fresh thalli were desiccated using silica gel and later kept frozen at -20 °C. Total DNA was extracted using the commercial EasyPure Plant Genomic DNA Kit (TransGen Biotech Corporation, Beijing), according to the manufacturer’s protocols. Two molecular markers, *rbc*L and COI-5P, were amplified using the primers and protocols described by Vis et al. (1998) and Saunders (2005). The PCR products with their amplification primers were sent to BGI Tech Corporation (Beijing, China) for sequencing on an ABI 3730XL sequencer. Sequences generated in this study were submitted to GenBank databases (Table 1). Additional related sequence data of the genus *Sheathia* and the outgroup taxa *Batrachospermum* were downloaded from GenBank (http://www.ncbi.nlm.nih.gov/) (Suppl. material 1).

The 63 *rbc*L and 49 COI-5P sequences were aligned using BioEdit v.7.2.5 (Hall 1999). The pairwise distances and nucleotide variations between sequences were calculated via MEGA 6.0 (Tamura et al. 2013). Subsequently, PhyloSuite v.1.2.2 (Zhang et al. 2020) was used to select the best-fit substitution models for single-gene datasets using ModelFinder based on the Bayesian Information Criterion (BIC). Following the determination of the best models for *rbc*L and COI-5P genes, Maximum Likelihood (ML) analysis was conducted using IQ-TREE v.1.6.8 (Nguyen et al. 2015), with node support assessed via the Shimodaira-Hasegawa test and the approximate likelihood ratio test (Guindon et al. 2010). Concurrently, Bayesian Inference (BI) analysis was performed using MrBayes v.3.2.6 (Ronquist et al. 2012). The analysis ran for 5,000,000 generations, sampling every 100 generations. The first 25% of trees were discarded as burn-in, and the analysis continued until the standard deviation of split frequencies fell below 0.01. Finally, the resulting phylogenetic trees were visualized and edited using FigTree v1.3.1 (http://tree.bio.ed.ac.uk/software/figtree/).

## Results

### Sequence information

The *rbc*L data matrix included 57 specimens of the genus *Sheathia* and 6 outgroup taxa, consisting of 1,207 nucleotides, of which 351 (29.08%) were variable and 297 (24.61%) were parsimony informative. The *rbc*L uncorrected *p*-distances among the six “Chantransia” isolates and other specimens of the genus *Sheathia* are listed in Suppl. material 2. Genetic divergences between the isolates CQ003, CQM21, CQM22, CQW23, and CQW24 (collected from Chongqing), and seven previously reported specimens of *S. dispersa* from China, Nepal, Japan, and USA ranged from 0% to 1%, corresponding to 0–12 base pair differences. In contrast, the genetic distance among these five isolates and other species within the genus *Sheathia* were 2% to 10%, corresponding to 25–115 bp. Furthermore, the pairwise distance between *S. shaoguanensis* sp. nov. (isolates GD247) and the other species of the genus *Sheathia* showed larger variance than among the intraspecific distance of this genus (4.7% VS 0.2%).

The COI-5P alignment of species was 567 bp, with 206 bp variable sites (among which 186 bp were parsimony informative sites). The pairwise distance among the “Chantransia” isolates collected in this study and other *Sheathia* taxa (Suppl. material 3) showed that isolates CQ003, CQM21, CQM22, CQW23, and CQW24 were closely related to five previously reported specimens of *S. dispersa* from China and USA; the only with COI-5P data. The mean *p*-distance among samples from China and USA was 1%. The intraspecific *p*-distances of species in this genus were 0–1%, whereas the interspecies *p*-distances of COI-5P sequences among the species of *Sheathia* were 3–15%. For the remaining isolate GD247, the pairwise distances with other Batrachospermales specimens also supported its unique taxonomic status: the *p*-distance between GD247 and the other species of *Sheathia* was 9.7%, therefore, much higher than the intraspecific distance within species of the genus (0–1%).

### Phylogenetic analyses and species delimitation

**Figure 1.**
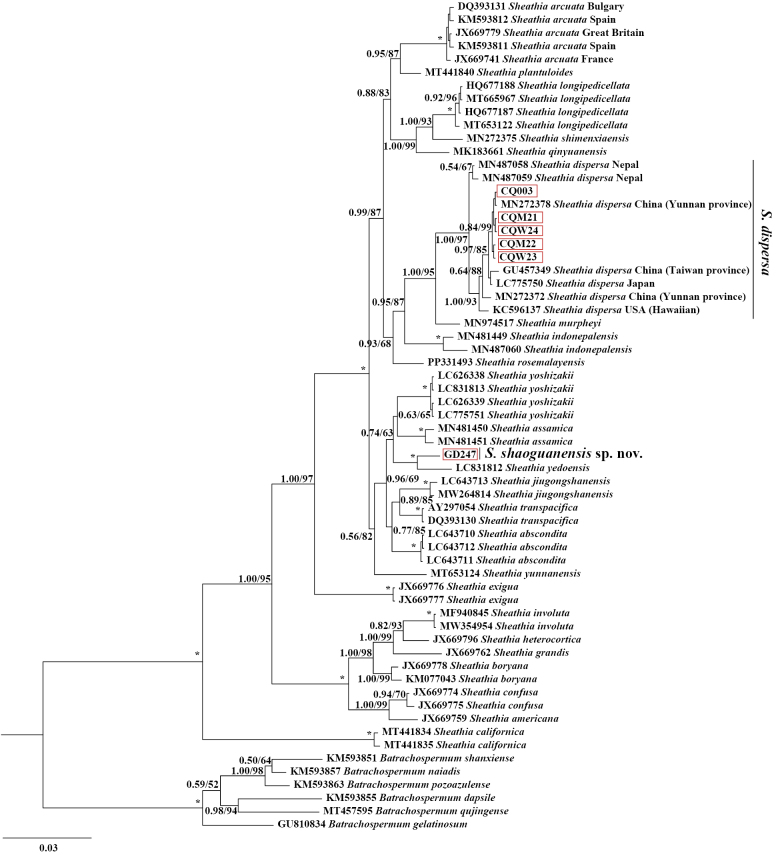
Bayesian inference tree based on the *rbc*L gene sequences. Bayesian posterior probabilities (>0.50) and ML bootstrap values (>50%) are shown at branches. All newly generated sequences in this study are indicated in red boxes. Asterisks on the nodes represent that the support values of Bayesian inference and Maximum Likelihood are both 100%.

**Figure 2.**
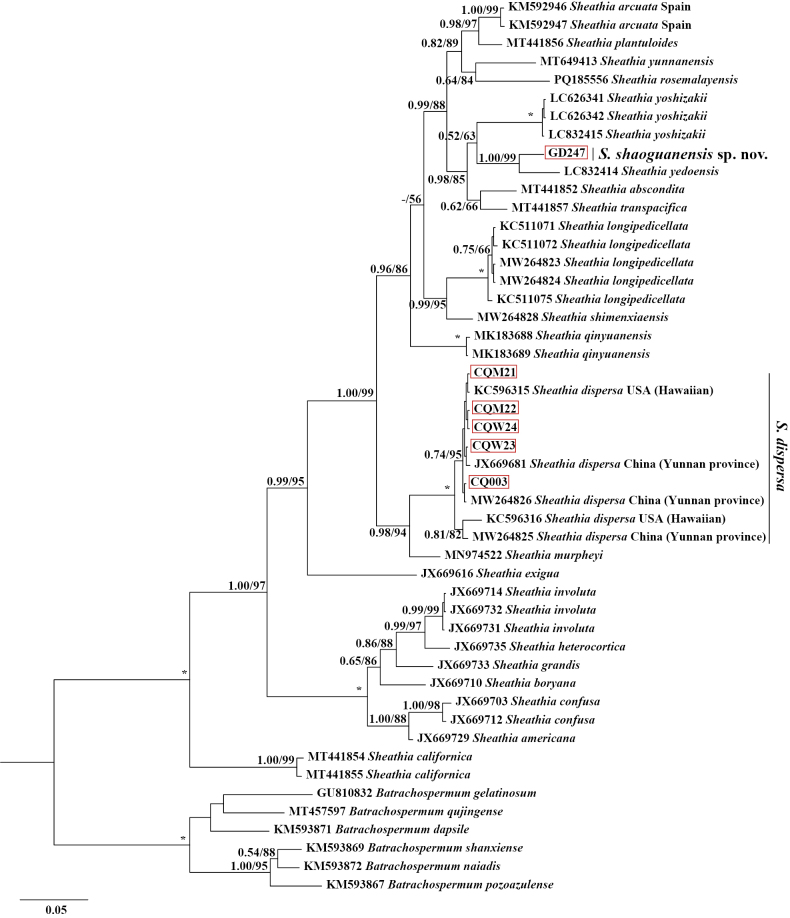
Bayesian inference tree based on the COI-5P gene sequences. Bayesian posterior probabilities (>0.50) and ML bootstrap values (>50%) are shown at branches. All newly generated sequences in this study are indicated in red boxes. Asterisks on the nodes represent that the support values of Bayesian inference and Maximum Likelihood are both 100%.

Both phylogenetic analyses (BI and ML) based on the *rbc*L and COI-5P alignments showed similar topological structures between the present species and its sister specimens, except that the main branches were arranged differently. Thus, only the BI trees are shown with the supporting values calculated from the two methods in Figs 1, 2. In the *rbc*L and COI-5P phylogenetic trees, the genus *Sheathia* was divided into four major clades. The six putative “Chantransia” isolates utilized in this study all belong to the first clade, which comprises seventeen distinct lineages. Within this first clade, except for a few species that were observed only in the “Chantransia” sporophyte generations (e.g., *S. jiugongshanensis* J.-F.Han, F.-R.Nan & S.L. Xie, *S. shimenxiaensis* J.-F.Han, F.-R.Nan & S.L.Xie, *S. qinyuanensis* J.-F.Han, F.-R.Nan & S. L.Xie, and *S. yedoensis* Kitayama & M.Suzuki), the remaining lineages lacked heterocortication.

In this clade, five isolates (CQ003, CQM21, CQM22, CQW23, and CQW24) collected from Chongqing were clustered with several specimens from China (Yunnan and Taiwan), Nepal, Japan and USA to form a large clade named *S. dispersa*, which had high support values (Fig. 1: 1.00/97; Fig. 2: 1.00/100). Similarly, in the phylogenetic trees, specimen “GD247”, collected from the Guangdong Grand Canyon in Shaoguan, Guangdong Province, formed a distinct clade. It was sister to the clade containing *S. yedoensis* from Japan (Fig. 1: 1.00/100; Fig. 2: 1.00/99). Although the two specimens shared a close phylogenetic relationship, pairwise genetic distances indicated that the distance between “GD247” and *S. yedoensis* (2%) was larger than the intraspecific genetic distance within species of the genus *Sheathia* (0–1%), supporting that they represent two distinct species and confirming the independent taxonomic status of “GD247” within the genus *Sheathia*.

### Morphological observations

**Figure 3.**
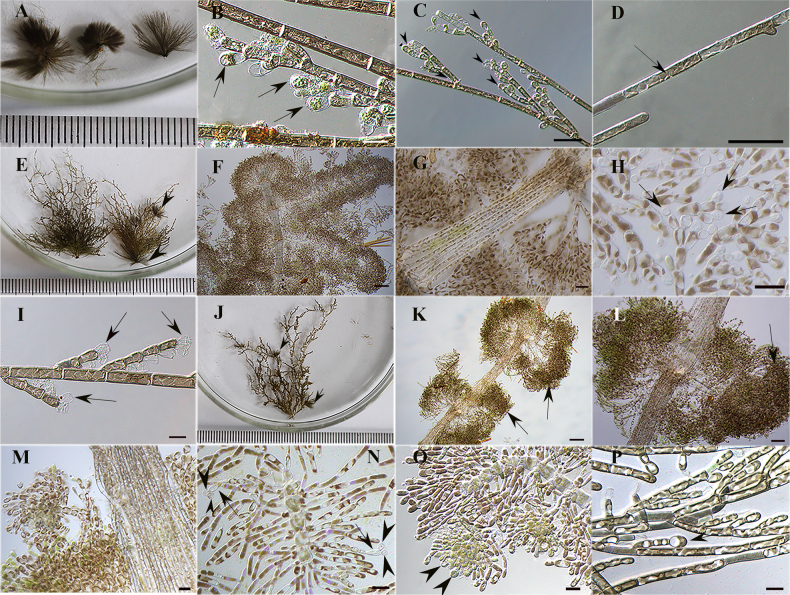
Morphological characters of *S. dispersa* investigated in this study. **A–D**. Isolate CQ003. **A**. Morphological observation of the tufts of erect filament, brownish in color; **B**. Monosporangial branch with ovoid monosporangia (arrows); **C**. Filament showing branch angle < 25° (arrow) and monosporangial branch with ovoid monosporangium (arrowheads); **D**. Cells have irregularly lobed Chromatoplast (arrow). **E–I**. Sample CQM21 (male plant) and habit of “Chantransia” isolate CQM22 with highly branched audouinelloid filaments twining around gametophyte of *S. dispersa*. **E**. Morphological characters of the male plants and their “Chantransia” stages; **F**. Spherical- to barrel-shaped, adjacent and well-developed whorls; **G**. Cortical cells containing only cylindrical cells; **H**. Spermatangia (arrows) on the tips of fascicle cells; **I**. Filament showing branch angle ≤ 25° and monosporangia (arrows). **J–P**. Sample CQW23 (female plant) and habit of “Chantransia” isolate CQW24 with highly branched audouinelloid filaments twining around gametophyte of *S. dispersa*. **J**. Morphological characters of the female plant and the “Chantransia” isolates; **K, L**. Separated and spherical whorls with carposporophytes (arrows) extending out of the whorls; **M**. Cortical cells containing only cylindrical cells; **N**. Carpogonia with clavate trichogynes (arrows) with attached spermatia (arrowheads); **O**. Morphological characters of carposporophytes and obovoidal carposporangia (arrowheads); **P**. Filament showing branch angle ≤ 25° and monosporangium (arrow). Scale bars: 20 μm (**G–I, M–P**); 100 μm (**F, K**); 50 μm (**C, D, L**).

**Figure 4.**
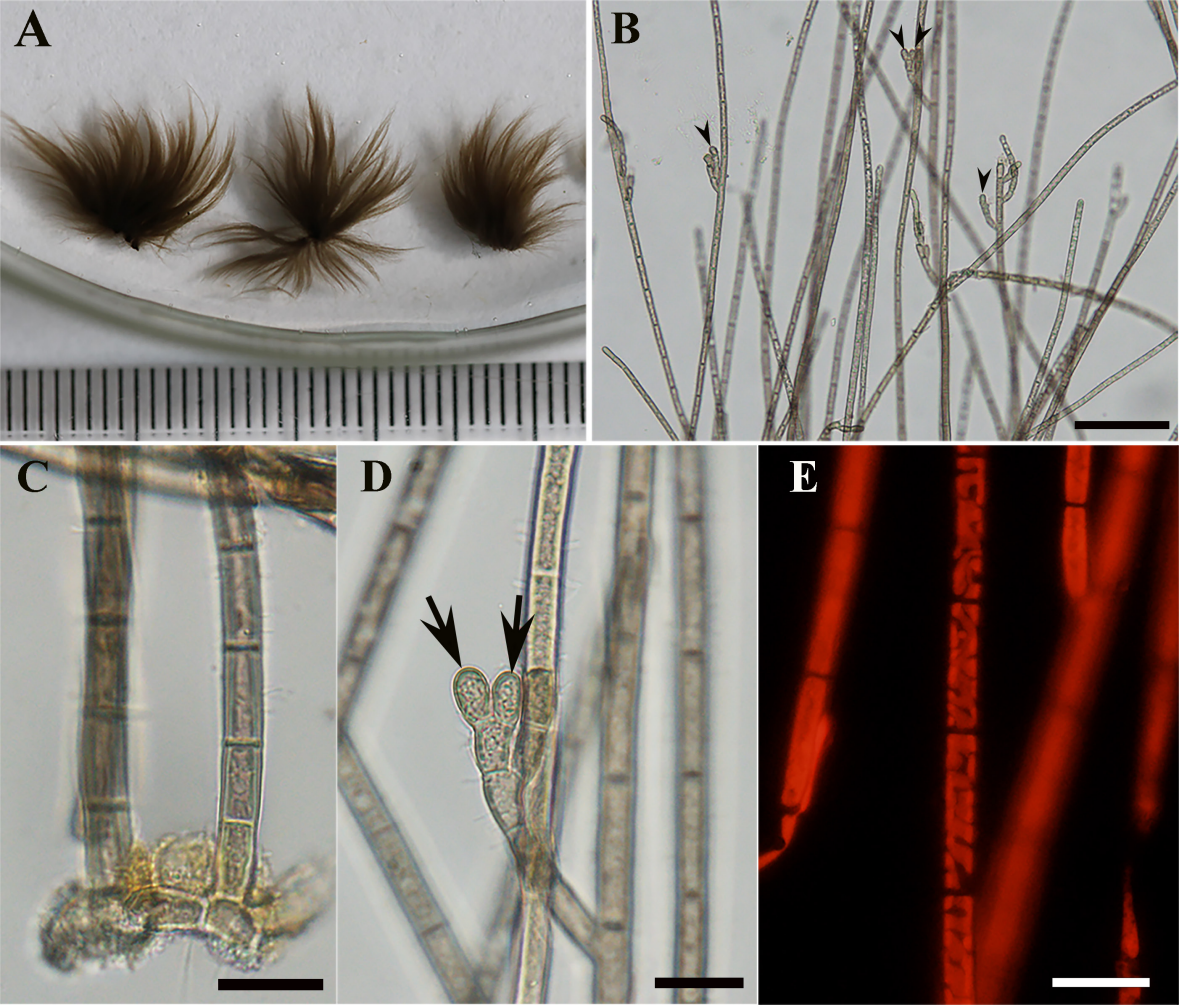
Morphological characters of *S. shaoguanensis* sp. nov. investigated in this study. **A**. Morphological observation of the tufts of erect filament, brownish in color; **B**. Erect filaments with monosporangia (arrowheads); **C**. Prostrate and erect systems, each cell of which containing a single parietal irregularly lobed chloroplast; **D**. Monosporangial branch with ovoid monosporangia (arrowheads); **E**. Cells have irregularly lobed Chromatoplast. Scale bars: 100 μm (**B**); 20 μm (**C–E**).

The morphological characteristics of the six specimens in this study were observed and measured as shown in Figs 3, 4 and Tables 2, 3. The two gametophyte specimens (CQM21 and CQW23) are in good agreement with the dimensions in the circumscription of *S. dispersa*. Of these, specimen CQM21 was identified as a male gametophyte, and CQW23 as a female gametophyte of *S. dispersa*. These two specimens have confluent or separated, barrel-shaped or spherical whorls, 409–709 µm in diameter. Cortication of the main axis with two or three layers of filaments, composed only of cylindrical cells. Furthermore, the spermatangia of the male gametophyte (CQM21) are spherical, terminal on primary fascicles, and 5.5–7.3 µm in diameter. As for the female gametophyte (CQW23), it is characterized as follows: purple to reddish with yellow-brownish hue; carposporophytes spherical, and exserted from the whorls, 103–353 μm in diameter; carposporangium 8.9–13.3 μm long and 5.6–6.7 μm in diameter; carpogonial branch erect and uncoiled with 3–5 cells; carpogonium 8.9–13.3 μm long, 5.6–6.7 μm in diameter with clavate trichogynes.

The four “Chantransia” isolates (CQ003, CQM22, CQW24, and GD247) were morphologically similar to *Audouinella pygmaea* (Kützing) Weber Bosse, whereas the vegetative cells and monosporangia of GD247 were smaller than those of the other three specimens. These four isolates had the following characteristics: brownish in color, forming small tufts on surfaces; thalli consisting of branched filaments with vegetative cells 5.3–17.8 µm in diameter and 22.1–77.8 µm in length; monosporangia obovoidal, in clusters or singly at branch tips; 6.3–13.3 µm in diameter and 8.4–21.1 µm in length.

## Taxonomic proposals

The genetic distance and phylogenetic analysis based on *rbc*L and COI-5P genes all supported the identification of a new species described below.

### Order Batrachospermales Pueschel & K.M.Cole, 1982

**Family Batrachospermaceae C.Agardh, 1824**

#### Sheathia shaoguanensis

Taxon classificationPlantaeBatrachospermalesBatrachospermaceae

J.F.Han & S.L.Xie sp. nov.

CF2D5A29-FF04-5504-A6AC-B5210236AB0D

Fig. 4 A–E

##### Description.

Known only from the “Chantransia” sporophyte generation. Plants usually hemispherical in outline, 3.6–9.8 mm high, bluish; composed of prostrate and erect filaments; lateral branches developing at an angle ≤ 25°; Vegetative cells of main branches cylindrical, 22.1–33.7 μm long and 5.3–7.4 μm in diameter. Monosporangia numerous, mostly grow on top of the 1–5 celled short branchlets, single or grouped, ovoidal, 8.4–13.7 μm long and 6.3–11.6 μm in diameter.

##### Diagnosis.

Diagnostic DNA sequence: *rbc*L and COI-5P (accession number: PZ319268 for *rbc*L and PZ321420 for COI-5P).

##### Type locality.

China, • Guangdong Province, Guangdong Grand Canyon (24°31'N,113°07'E), on the surface of a stone in flowing water.

##### Holotype here designated.

GKD-GD20247 (Fig. 4), Dried material prepared from reference strain GD247, Shanxi Vocational University of Engineering Science and Technology Herbarium (GKD), Shanxi Vocational University of Engineering Science and Technology, Jinzhong, Shanxi Province, China. November 2020 by Jin-fen Han and Kunpeng Fang.

##### Paratype.

China • Guangdong Province, Shaoguan, GKD-GD20248, Preserved samples in 4% formalin solution, prepared from reference strain GD248, Shanxi Vocational University of Engineering Science and Technology Herbarium (GKD), Shanxi Vocational University of Engineering Science and Technology, Jinzhong, Shanxi Province, China.

##### Etymology.

The species epithet refers to the type locality (Shaoguan, China).

##### Authentic strain.

GKD-GD20248.

##### Registration.

http://phycobank.org/107386.

## Discussion

Previous studies have demonstrated that the genus *Sheathia* is a monophyletic group within the Batrachospermales, ‌with a triphasic life cycle (Entwisle et al. 2009; Salomaki et al. 2014; Han et al. 2018). Since the carposporophyte grows on the gametophyte, we collectively refer to both the gametophyte and the gametophyte bearing carposporophyte as gametophyte samples‌ in this study. The specimens collected in this study included both gametophyte and “Chantransia” stage individuals. However, distinguishing *Sheathia* species is challenging due to their highly conserved morphological structures. Furthermore, the “Chantransia” stage of the genus *Sheathia* closely resembles the genus *Audouinella* in morphology (Han et al. 2020; Han et al. 2021; Kitayama and Suzuki 2024). Consequently, relying solely on morphological observation often leads to the misidentification of the “Chantransia” stage as *Audouinella* (Pueschel et al. 2000; Chiasson et al. 2005), which poses significant difficulties for the taxonomy and identification of freshwater red algae.

In recent years, with the rapid development of molecular techniques, molecular-assisted tools have played a pivotal role not only in evaluating phylogenetic positions but also in providing novel insights into delimiting the taxonomic status of morphologically similar specimens. Particularly within the genus *Sheathia*, numerous new species have been described based on the *rbc*L, *psb*A, UPA, and COI-5P genes, such as: *S. qinyuanensis*, *S. jiugongshanensis*, *S. shimenxiaensis*, *S. plantuloides* and *S. yedoensis* (Han et al. 2020; Han et al. 2021; Kitayama and Suzuki 2024).

In this study, phylogenetic analyses were conducted on gametophytic and “Chantransia” specimens collected from various regions of China, based on the *rbc*L and COI-5P genes. The results consistently indicated that the five isolates from Chongqing Municipality (CQ003, CQM21, CQM22, CQW23, and CQW24) belong to *S. dispersa*. Morphological observations (Fig. 3) confirmed that CQM21 and CQW23 were gametophytes, while the remaining specimens were “Chantransia” stages. Numerous studies have shown that most freshwater red algae grow attached in relatively stable, low-flow, and small-scale water bodies such as springs, wells, and streams, and are rarely found in large rivers and lakes. Due to their fragile adaptability to the environment, they are typically restricted to specific microhabitats—for instance, not extending beyond the spring source in springs, or being confined to a specific river section, usually within 10 meters, in streams. This results in relatively isolated populations with little genetic exchange.

**Figure 5.**
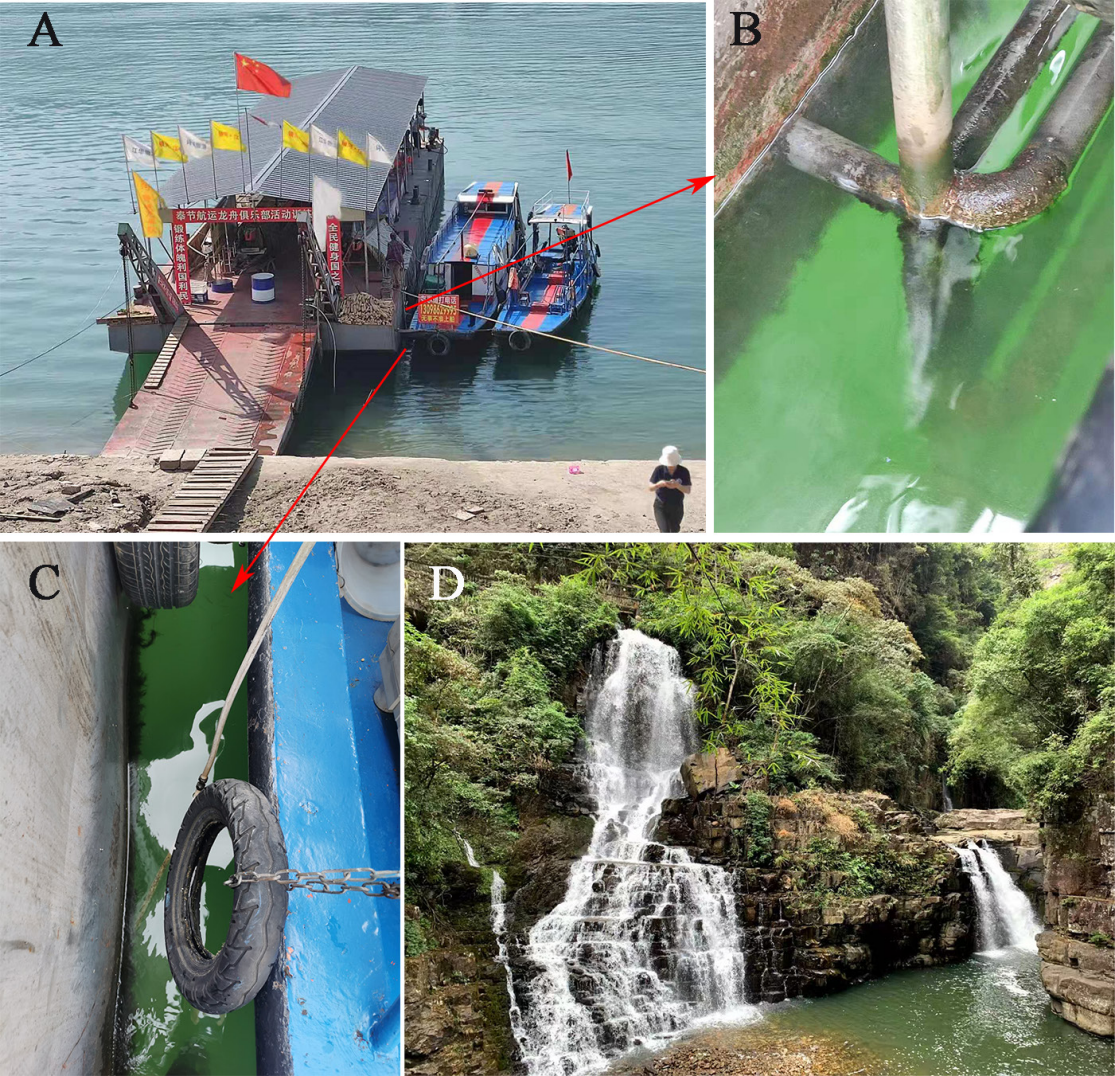
Habitat of *Sheathia dispersa* and *S. shaoguanensis* sp. nov. **A**. Habitat of *Sheathia dispersa* utilized in this study; **B**. Specimen CQ003 collected from metal ladder on embankments along the Yangtze River in Fengjie County, Chongqing; **C**. Sampling site of specimens CQM21, CQM22, CQW23 and CQW24: cement wall of the embankment along the Yangtze River in Fengjie County, Chongqing City; **D**. Habitat of *S. shaoguanensis* (GD247).

Notably, the five specimens collected in Chongqing in this study were found in a unique habitat, attached to metal stairs and the cement wall of the embankment along the Yangtze River (Fig. 5A–C). As the longest river in China and the third longest in the world, the Yangtze River spans approximately 6,300 kilometers with an annual runoff exceeding 960 billion cubic meters. Its vast drainage area, immense water volume, and complex, dynamic hydrological conditions create a distinctive riverine ecosystem. Consequently, the habitat of these specimens differs significantly from that of most previously reported *Sheathia* species. The Yangtze River far exceeds the small water bodies traditionally inhabited by this genus in both scale and discharge, and its environmental stability is much lower. Characterized by rapid currents, significant seasonal water-level fluctuations, high sediment loads, and complex nutrient dynamics, these habitat features stand in stark contrast to the relatively stagnant, clear, and stable conditions of small water bodies. However, the five specimens discovered in this study were able to complete their growth and development in this environment, with two even reaching the gametophyte stage. This suggests that the species possesses a stronger adaptability to heterogeneous habitats than previously recognized, thereby expanding our understanding of the habitat range of *Sheathia* species.

Since we also found other freshwater red algae specimens in the “Chantransia” stage from nearby mountain streams, combined with the actual local geographical environment, we hypothesize that these *S. dispersa* populations were likely transported to the embankments via streams flowing from the mountains on both banks into the Yangtze River. Previously, *S. dispersa* was only reported from the Hawaiian Islands, Nepal, India, Japan, and the Yunnan province and the island of Taiwan of China (Necchi et al. 2019). Therefore, this study expands the global known distribution of *S. dispersa* and extends its distribution in China to three locations: Taiwan, Yunnan, and Chongqing.

Similarly, phylogenetic trees based on *rbc*L and COI-5P genes, as well as *p*-distances analysis, indicated that specimen GD247 represents a new species of the genus *Sheathia*. However, since GD247 was the “Chantransia” of *S. shaoguanensis* sp. nov. and no gametophytes were found in the surrounding habitat, morphological identification remains currently unfeasible. Further collection of gametophyte specimens and a comprehensive analysis of morphological traits are required. In both phylogenetic trees, GD247 forms a well-supported sister relationship with *S. yedoensis* from Japan, indicating a close phylogenetic affinity. According to Kitayama and Suzuki (2024), *S. yedoensis* was also described based on the “Chantransia” sporophyte stage. It is characterized by the following features: the thalli are reddish to dark brown, reaching heights of up to 4 mm. The erect filaments are uniseriate, irregularly and sparsely branched at angles of 12°–21°. Vegetative cells are cylindrical, measuring 8–10 µm in diameter and 14–57 µm in length. Monosporangia are ovoid to ellipsoidal (7–11 µm in diameter, 10–14 µm in length) and are borne terminally or laterally on short, 2–4-celled laterals of the erect filaments. Comparative morphological analysis reveals overlapping features between these two specimens in terms of thallus height, vegetative cells, and unispore size, suggesting that they cannot be reliably distinguished based on morpho-anatomical characteristics alone.

In the freshwater red algal genus *Sheathia*, species display two distinct cortical structure patterns. One possess heterocortication, where both bulbous and cylindrical cells cover the main axis, while the other have homocortication, made up exclusively of cylindrical cells (Salomaki et al. 2014). All species within the so called *Sheathia arcuata* complex are homocorticated (Necchi et al. 2019; Vis et al. 2020). Phylogenetic trees based on *rbc*L and COI-5P genes showed that the six specimens collected in this study clustered within the *S. arcuata* complex, indicating that the two species treated in this study belong to the large clade of cryptic species with homocortication. Thus, despite the absence of gametophytes for the newly described species *S. shaoguanensis* sp. nov., it can be inferred that its cortication of the main axis might consist exclusively of cylindrical cells.

In summary, although the majority of specimens collected in this study were in the “Chantransia” stage, with only a few in the gametophyte stage, molecular methods revealed a rich species diversity among these “Chantransia” isolates, including a new species of the genus *Sheathia*. This suggests that despite their simple morphology and frequent confusion with *Audouinella*, the “Chantransia” stages harbor a hidden diversity, which is likely linked to their strong stress resistance. Previous studies support this view. For instance, Chiasson et al. (2005) observed that environmental physicochemical parameters could inhibit the production of gametophytes in filamentous stages during the same season. Furthermore, Zucchi and Necchi (2003) and Chiasson et al. (2007) reported that filaments could persist in streams where gametophytes were never observed. Additionally, the new species *Thorea quisqueyana* was described based solely on filamentous specimens.

## Conclusion

In this study, we conducted a systematic analysis of six specimens collected from South China based on morphological characteristics and genetic data (*rbc*L and COI-5P). Morphological observations indicated that the “Chantransia” isolates were similar to *A. pygmaea*, while the gametophyte stage samples were highly consistent with the description of *S. dispersa*. However, molecular phylogenetic analysis further clarified their taxonomic status: the five specimens collected from the levees along the Yangtze River in Fengjie County, Chongqing, were confirmed as the gametophyte and “Chantransia” stages of *S. dispersa*, whereas the isolate GD247 collected from Guangdong Province was identified as a new species of the genus *Sheathia*—*S. shaoguanensis*. This finding not only enriches the species diversity of the genus *Sheathia* in China but also expands our understanding of the habitat range of these taxa, strongly supporting the view that “*A. pygmaea*” is actually a morphological assemblage of the “Chantransia” stages of multiple species. These results provide new evidence for elucidating the correspondence of life history stages in freshwater red algae and supplement the distribution records of *Sheathia* in South China. Future collection and analysis of specimens from broader regions will help to further clarify the species boundaries and phylogenetic relationships of this group, ultimately improving the species catalog of freshwater red algae in China.

## Supplementary Material

XML Treatment for Sheathia shaoguanensis

## Additional information

### Artificial Intelligence (AI) use

The authors accept full responsibility for the content of the manuscript, including the disclosure of any use of AI.

No AI tools were used in the preparation of this manuscript.

### Author ORCIDs

J.-f. Han https://orcid.org/0000-0002-3169-6253

X. Wang https://orcid.org/0009-0006-9793-2788

B. Yin https://orcid.org/0009-0004-2877-0901

S.-l. Xie https://orcid.org/0000-0003-2349-2071

### Data availability

All of the data that support the findings of this study are available in the main text or Supplementary Information.

## Supplementary materials

Supplementary material 1 Specimen information of sequences downloaded from the GenBank database Data type pdf https://binary.pensoft.net/file/1709056This dataset is made available under the Open Database License (http://opendatacommons.org/licenses/odbl/1.0/). The Open Database License (ODbL) is a license agreement intended to allow users to freely share, modify, and use this Dataset while maintaining this same freedom for others, provided that the original source and author(s) are credited. Jin-fen Han, Xin Wang, Bin Yin, Shu-lian Xie

Supplementary material 2 Pairwise distance (lower-left matrix) and number of nucleotide variance (upper-right matrix) of *rbc*L sequence among the taxa in this study Data type xls https://binary.pensoft.net/file/1709057This dataset is made available under the Open Database License (http://opendatacommons.org/licenses/odbl/1.0/). The Open Database License (ODbL) is a license agreement intended to allow users to freely share, modify, and use this Dataset while maintaining this same freedom for others, provided that the original source and author(s) are credited. Jin-fen Han, Xin Wang, Bin Yin, Shu-lian Xie

Supplementary material 3 Pairwise distance (lower-left matrix) and number of nucleotide variance (upper-right matrix) of COI-5P sequence among the taxa in this study Data type xls https://binary.pensoft.net/file/1709058This dataset is made available under the Open Database License (http://opendatacommons.org/licenses/odbl/1.0/). The Open Database License (ODbL) is a license agreement intended to allow users to freely share, modify, and use this Dataset while maintaining this same freedom for others, provided that the original source and author(s) are credited. Jin-fen Han, Xin Wang, Bin Yin, Shu-lian Xie
