# Supplementary material for: Sheathia shaoguanensis sp. nov. (Batrachospermales, Rhodophyta), a new freshwater red algal species from South China
Source: PhytoKeys. 2026 Jul 6;277:25–40. doi: 10.3897/phytokeys.277.198746 (PMC13366045; doi:10.3897/phytokeys.277.198746)
Supplement: Supplementary material 1 — Specimen information of sequences downloaded from the GenBank database [file phytokeys-277-025_article-198746__-s001.pdf]

Sheathia shaoguanensis sp. nov. (Batrachospermales, Rhodophy): a new freshwater red algal species from South China

Jin-fen Han[1], Xin Wang[1], Bin Yin[2], Shu-lian Xie[3]

1 *Collega of Architecture and Design, Shanxi Vocational University of Engineering Science and Technology, Jinzhong 030619, China*

2 *School of Engineering Management, Shanxi Vocational University of Engineering Science and Technology, Jinzhong 030619, China*

3 *School of Life Science, Shanxi Key Laboratory for Research and Development of Regional Plants, Shanxi University, Taiyuan 030006, China*

Corresponding author: Jin-fen Han (*hanjinfen@sxgkd.edu.cn*)

Supplementary Tables

Supplementary Table S1 Specimen information of sequences downloaded from the GenBank database. "–" denotes no related information for the specimen.

Species	*rbc*L accession No.	Reference	COI accession No.	Reference
Batrachospermum dapsile	KM593855	Unpublished	KM593871	Unpublished
B. gelatinosum	GU810834	House et al. 2010	GU810832	House et al. 2010
B. naiadis	KM593857	Unpublished	KM593872	Unpublished
B. pozoazulense	KM593863	Unpublished	KM593867	Unpublished
B.qujingense	MT457595	Fang et al. 2020	MT457597	Fang et al. 2020
B. shanxiense	–	–	KM593869	Unpublished
Sheathia arcuata	DQ393131	Salomaki et al. 2014	–	–
S. arcuata	JX669741	Salomaki et al. 2014	KM592946	unpublished
S. arcuata	KM593812	Chapuis 2016	KM592947	unpublished
S. arcuata	JX669779	Salomaki et al. 2014	–	–
S. arcuata	KM593811	Chapuis 2016	–	–
S. boryana	JX669778	Salomaki et al. 2014	JX669710	Salomaki et al. 2014
S. boryana	KM077043	Abdelahad et al. 2015	–	–
S. plantuloides	MT441840	Vis et al. 2020	MT441856	Vis et al. 2020
S. longipedicellata	HQ677188	Li et al. 2010	KC511071	Ji et al. 2014
S. longipedicellata	MT665967	Fang et al. 2022	KC511072	Ji et al. 2014
S.	HQ677187	Li et al. 2010	MW264823	Han et al. 2022

longipedicellata				
S. longipedicellata	MT653122	unpublished	MW264824	Han et al.. 2022
S. longipedicellata	–	–	KC511075	Unpublished
S. shimenxiaensis	MN272375	Han et al. 2020	MW264828	Han et al.. 2022
S. qinyuanensis	MK183661	Han et al. 2020	MK183688	Han et al. 2021
S. dispersa	MN487058	Necchi et al., 2019	KC596315	Unpublished
S. dispersa	MN487059	Necchi et al. 2019	JX669681	Salomaki et al. 2014
S. dispersa	MN272378	Han et al. 2020	MW264826	Han et al.. 2022
S. dispersa	GU457349	Vis et al. 2010	KC596316	Carlile and Sherwood 2013
S. dispersa	LC775750	Kitayama and Suzuki 2024[a]	MW264825	Han et al. 2022
S. dispersa	MN272372	Han et al. 2020	–	–
S. dispersa	KC596137	Carlile and Sherwood 2013	–	–
S. murpheyi	MN974517	Szinte et al. 2020	MN974522	Szinte et al., 2020
S. indonepalensis	MN481449	Necchi et al. 2019	–	–
S. indonepalensis	MN487060	Necchi et al. 2019	–	–
S. rosemalayensis	PP331493	Jayalakshmi and John 2025	PQ185556	Jayalakshmi and John 2025
S. yoshizakii	LC626338	Suzuki and Kitayama 2021	LC626341	Suzuki and Kitayama 2021
S. yoshizakii	LC831813	Kitayama and Suzuki 2024[b]	LC626342	Suzuki and Kitayama 2021
S. yoshizakii	LC626339	Suzuki and Kitayama 2021	LC832415	Kitayama and Suzuki 2024[b]
S. yoshizakii	LC775751	Kitayama and Suzuki 2024[a]	–	–
S. assamica	MN481450	Necchi et al. 2019	–	–
S. assamica	MN481451	Necchi et al. 2019	–	–
S. yedoensis	LC831812	Kitayama and Suzuki 2024[b]	LC832414	Kitayama and Suzuki 2024[b]
S. jiugongshanensis	LC643713	Kitayama et al. 2010	–	–

S. jiugongshanensis	MW264814	Han et al. 2022	–	–
S. transpacifica	AY297054	Vis et al. 2010	MT441857	Vis et al., 2020
S. transpacifica	DQ393130	Vis et al. 2010	–	–
S. abscondita	LC643710	Kitayama et al. 2010	–	–
S. abscondita	LC643712	Kitayama et al. 2010	MT441852	Vis et al., 2020
S. abscondita	LC643711	Kitayama et al. 2010	–	–
S. yunnanensis	MT653124	Fang et al. 2022	MT649413	Fang et al. 2022
S. exigua	JX669776	Salomaki et al. 2014	JX669616	Salomaki et al. 2014
S. exigua	JX669777	Salomaki et al. 2014	–	–
S. involuta	MF940845	Unpublished	JX669714	Salomaki et al. 2014
S. involuta	MW354954	Unpublished	JX669732	Salomaki et al. 2014
S. involuta	–	–	JX669731	Salomaki et al. 2014
S. heterocortica	JX669796	Salomaki et al. 2014	JX669735	Salomaki et al. 2014
S. grandis	JX669762	Salomaki et al. 2014	JX669733	Salomaki et al. 2014
S. confusa	JX669774	Salomaki et al. 2014	JX669703	Salomaki et al. 2014
S. confusa	JX669775	Salomaki et al. 2014	JX669712	Salomaki et al. 2014
S. americana	JX669759	Salomaki et al. 2014	JX669729	Salomaki et al. 2014
S. californica	MT441834	Vis et al. 2020	MT441854	Vis et al., 2020
S. californica	MT441835	Vis et al. 2020	MT441855	Vis et al., 2020

References

Abdelahad N, Bolpagni R, Jona Lasinio G, Vis ML, Amadio C, Laini A, Keil EJ (2015) Distribution, morphology and ecological niche of *Batrachospermum* and *Sheathia* species (Batrachospermales, Rhodophyta) in the fontanili of the Po plain (Northern Italy). European Journal of Phycology 50(3): 318-329. https://doi.org/10.1080/09670262.2015.1055592.

Carlile AL, Sherwood AR. (2013) Phylogenetic affinities and distribution of the Hawaiian freshwater red algae (Rhodophyta). Phycologia 52(3): 309-319. https://doi.org/10.2216/12-097.1.

Chapuis IS. (2016) Batrachospermales (Rhodophyta) from the Iberian Peninsula and

the Balearic Islands: diversity and phylogeny. Ph.D. Dissertation. Granada: Universidad de Granada.
Fang KP, Nan FR, Feng J, Lv JP, Liu Q, Liu XD, Xie SL (2020) *Batrachospermum qujingense* (Batrachospermales, Rhodophyta), a new freshwater red algal species from Southwest China. Phytotaxa 461 (1): 1–11. https://doi.org/10.11646/phytotaxa.461.1.1
Fang KP, Nan FR, Feng J, Lv JP, Liu Q, Liu XD, Xie SL (2022). *Sheathia yunnanensis*, a new species of freshwater red alga (Rhodophyta: Batrachospermales) from Yunnan, China. Nordic Journal of Botany 2022(5): e03476. https://doi.org/10.1111/njb.03476
Han JF, Nan FR, Feng J, Lü, JP, Liu, Q, Liu, XD, Xie, SL (2020) Affinities of four freshwater putative "Chantransia" stages (Rhodophyta) in Southern China from molecular and morphological data. Phytotaxa 441 (1): 47–59. https://doi.org/10.11646/phytotaxa.441.1.4
Han JF, Nan FR, Feng J, Lü, JP, Liu, Q, Liu, XD, Xie, SL (2021) Affinities of freshwater "Chantransia" stage algae (Rhodophyta) from China based on molecular and morphological analyses. Journal of Oceanology and Limnology 39(3): 1063-1076. https://doi.org/10.1007/s00343-020-0114-6
Han JF, Nan FR, Feng J, Lü, JP, Liu, Q, Liu, XD, Xie, SL (2022). Phylogenetic, evolutionary, and biogeographic origin of the genus Sheathia (Batrachospermales, Rhodophyta). Journal of Oceanology and Limnology 40(2): 729-744. https://doi.org/10.1007/s00343-021-1075-0
House DL, Vandenbroek AM, Vis ML (2010) Intraspecific genetic variation of *Batrachospermum gelatinosum* (Batrachospermales, Rhodophyta) in eastern North America. Phycologia 49(5): 501-507. https://doi.org/10.2216/09-104.1
Jayalakshmi P.S, John J (2025) *Sheathia rosemalayensis* (Batrachospermales, Rhodophyta), a new species of freshwater red algae from the Western Ghats, India. Phycologia 64(1), 84-90. https://doi.org/10.1080/00318884.2025.2473126
Ji L, XIE SL, Feng J, Chen L, Wang J (2014) Molecular systematics of four endemic Batrachospermaceae (R hodophyta) species in China with multilocus data. Journal of Systematics and Evolution 52(1): 92-100. https://doi.org/10.1111/jse.12058
Kitayama T, Suzuki M (2024a) Phenology of *Sheathia abscondita* Stancheva, Sheath & ML Vis (Batrachospermaceae, Rhodophyta) in Hokkaido, Japan. Bulletin of the National Museum of Nature and Science. Series B, Botany 50(3): 93-103. https://doi.org/10.50826/bnmnsbot.50.3_93
Kitayama T, Suzuki M (2024b) *Sheathia yedoensis*, a New Species of the Freshwater Red Alga (Batrachospermaceae, Rhodophyta) from Kitanomaru Park, Adjacent to the Imperial Palace, Tokyo, Japan. Bulletin of the National Museum of Nature and Science. Series B, Botany 50(4): 131–140. https://doi.org/10.50826/bnmnsbot.50.4_131
Kitayama T, Kiyosue Y, Kozono J, Hanyuda T, Suzuki M (2021) First Record of *Sheathia abscondita* Stancheva, Sheath & ML Vis (Batrachospermaceae, Rhodophyta) from Japan. Bulletin of the National Museum of Nature and Science. Series B, Botany 47(4): 175-182. https://doi.org/DOI:10.50826/bnmnsbot.47.4_175

Li Q, Ji L, Xie SL. (2010) Phylogenetic analysis of Batrachospemales (Florideophyceae, Rhodophyta) based on chloroplast *rbc*L sequences. Acta Hydrobiologica Sinica 34(1): 20-28. https://doi.org/10.3724/sp.j.1035.2010.00020. (in Chinese with English abstract)

Necchi O, West JA, Ganesan EK, Yasmin F, Rai SK, Rossignolo NL. (2019) Diversity of the genus *Sheathia* (Batrachospermales, Rhodophyta) in northest India and east Nepal. Algae. An International Journal of Algal Research 34(4): 277-288. https://doi.org/10.4490/algae.2019.34.10.30.

Salomaki ED, Kwandrans J, Eloranta P, Vis ML (2014) Molecular and morphological evidence for Sheathia gen. nov. (Batrachospermales, Rhodophyta) and three new species. Journal of Phycology 50: 526–542. https://doi.org/10.1111/jpy.12179

Suzuki M, Kitayama T (2021) A new species of the genus *Sheathia* (Batrachospermaceae, Rhodophyta) from Japan. Phycologia, 60(4), 368-374. https://doi.org/10.1080/00318884.2021.1946643

Szinte AL, Taylor JC, Abosede AT, Vis ML. (2020) Current status of freshwater red algal diversity (Rhodophyta) of the African continent including description of new taxa (Batrachospermales). Phycologia 59(3): 187-199. https://doi.org/10.1080/00318884.2020.1732149.

Vis ML, Tiwari S, Evans JR, Stancheva R, Sheath RG, Kennedy B, Lee J, Eloranta P (2020) Revealing hidden diversity in the *Sheathia arcuata* morphospecies (Batrachospermales, Rhodophyta) including four new species. Algae 35(3): 213-224. https://doi.org/10.4490/algae.2020.35.8.31.

Vis ML, Feng J, Chiasson WB, Xie SL, Stancheva R, Entwisle TJ, Chou JY, Wang WL (2010) Investigation of the molecular and morphological variability in *Batrachospermum arcuatum* (Batrachospermales, Rhodophyta) from geographically distant locations. Phycologia 49(6): 545-553. https://doi.org/10.2216/10-04.1.
